# Longitudinal Ultrasonic Vibration-Assisted Planing Method for Processing Micro-Pyramid Arrays

**DOI:** 10.3390/mi15070923

**Published:** 2024-07-19

**Authors:** Jiashun Gao, Zhilong Xu, Bicheng Guo, Yu Lei, Guang Yang

**Affiliations:** 1 Xiamen Ocean Vocational College, Xiamen 361000, China; gaoshunjia@163.com (J.G.); guangyangxmoc@163.com (G.Y.); 2 School of Marine Engineering, Jimei University, Xiamen 361000, China; 3 School of Marine Equipment and Mechanical Engineering, Jimei University, Xiamen 361000, China; guobicheng0306@163.com; 4 Institute of Manufacturing Engineering, Huaqiao University, Xiamen 361000, China; 13886568044@163.com

**Keywords:** MPA, longitudinal ultrasonic vibration-assisted planing, surface roughness, edge burr, corrugated surface

## Abstract

Micro-pyramid copper molds are critical components in the preparation of high-precision optical elements, such as light-trapping films and reflective films. Their surfaces are composed of micro-pyramid arrays (MPAs). The surface roughness and edge burrs of MPAs seriously affect the optical properties of optical elements. To reduce the surface roughness, as well as the sizes of the edge burrs, the longitudinal ultrasonic vibration-assisted planing (LUVP) method for processing MPAs was developed during this study. In addition, an experiment was conducted to compare the precision planing and LUVP methods of MPA generation. The results show that the tool nose amplitude of the LUVP experimental platform constructed during this study was 3.3 μm, and that the operating frequency was 19.85 kHz. An MPA processed by LUVP had a smaller surface roughness than that of an MPA produced by precision planing; it also had fewer and smaller edge burrs, and there was slightly less diamond tool wear. The MPA cut using the LUVP method had no corrugation on its surface. This research lays a foundation for developing higher-precision micro-pyramid plastic films.

## 1. Introduction

**Figure 1:**
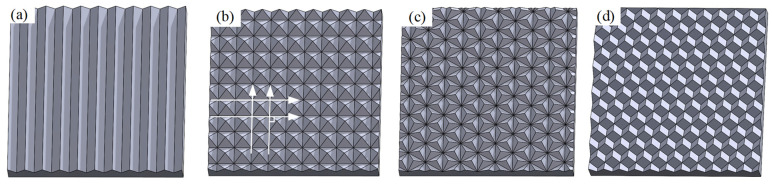
Micro-texture arrays with four different structures: (**a**) micro-V-shaped slot array, (**b**) MPA MPAs consist of perpendicular V-shaped slots, (**c**) micro-triangular pyramid array, and (**d**) full-cube pyramid array.

Micro-textured plastic films, because of their excellent refraction and reflection properties, are used in many different optical elements, such as light-trapping and reflective films [1,2,3,4]. Micro-textured copper molds are critical components in the micro-textured plastic film preparation process, and their surfaces are composed of micro-texture arrays [5]. Existing types of micro-texture arrays include micro-V-slot arrays [6], micro-pyramid arrays (MPAs) [7], micro-triangular pyramid arrays [8], and full-cube pyramid arrays [9], as shown in Figure 1. MPAs consist of perpendicular V-shaped slots, as shown in Figure 1b. They are widely used because of their excellent optical properties and simple processing technology. MPA machining methods include fly-cutting [10], grinding [7,11], and planing [12]. Compared with the grinding and fly-cutting methods, the planing method does not require the cutting tool to rotate, thereby eliminating machine spindle rotation error and simplifying the tool setting process. Planing is commonly used because it is inexpensive and is highly efficient.

Scholars have conducted meaningful research regarding MPA planing methods. Some scholars have used single-crystal diamond tools to perform MPA precision planing. They found that this method produces clear outlines and a good surface quality. However, due to the low cutting speeds and large cutting forces associated with this process, plastic deformation of the material was obvious and edge burrs were readily formed [13,14,15]. Edge burrs significantly affect the properties of optical components. To remove edge burrs, some scholars cut MPAs using the elliptic ultrasonic vibration-assisted planing method. Because the actual cutting speeds during ultrasonic planing processes are large and the cutting forces are small, only a small amount of edge burr residue is produced. However, it is difficult to prevent the emergence of wavy features on the MPA surfaces, and these features affect the surface roughness of the workpieces [16,17]. Some scholars also observed wavy features on the surfaces of workpieces processed by longitudinal torsion ultrasonic vibration-assisted cutting and three-dimensional ultrasonic vibration-assisted cutting [18,19]. Therefore, when using ultrasonic vibration-assisted planing to process MPAs, it is crucial to reduce the surface roughness and prevent the formation of wavy features.

To prevent the formation of wavy surface features that affect the surface quality when an MPA is planed with ultrasonic vibration assistance, it is necessary to ensure that the vibration direction is straight and parallel to the feed direction. Longitudinal ultrasonic vibration-assisted cutting (LUVC) can achieve linear vibration motion that is parallel to the feed direction. Scholars have conducted meaningful investigations into LUVC. Some scholars adopted longitudinal ultrasonic vibration-assisted milling technology to improve the machinability and surface quality of a titanium alloy (Ti-6Al-4V) and carbon fiber-reinforced silicon carbide matrix (C/SiC) composites [20,21]. Other scholars adopted longitudinal ultrasonic vibration-assisted drilling technology to drill holes into aviation parts made of carbon fiber-reinforced polymers; they achieved good results, thereby proving the effectiveness of longitudinal ultrasonic vibration-assisted drilling technology [22,23]. Some scholars used longitudinal ultrasonic vibration-assisted grinding to process alumina ceramics and continuous carbon fiber-reinforced silicon carbide matrix composites; they conducted thorough research regarding the process parameters and obtained good processing results [24,25,26]. The studies discussed above adopted LUVC technology; in these processes, the vibration direction was parallel to the feed direction and burrs were removed effectively. However, the LUVP method of producing MPAs has yet to be studied.

This paper proposes the LUVP method for processing MPAs and compares it with the MPA precision planing method. First, an LUVP device was designed and an associated modal analysis was conducted. Next, an LUVP experimental platform for MPA production was constructed according to the machining requirements and the vibration characteristics of the tool nose were tested. Then, the experimental results were analyzed and MPA precision planing results were compared with those of the LUVP method. Finally, a combination of theory and experiment was used to reveal the characteristics of the LUVP processing of MPAs, and the feasibility of the technique was verified. This study lays a foundation for the development of higher-precision micro-pyramid plastic films.

## 2. Methods and Materials

### 2.1. Design and Validation of the LUVP Device

#### 2.1.1. Design of the LUVP Device

The LUVP device had a transducer resonant frequency, *f*, of 20 kHz. A PZT8 piezoelectric ceramic plate with an outer circle diameter, *D*_0_, of 60 mm, a center hole diameter, *d*_0_, of 20 mm, a thickness, *l*_0_, of 6 mm, and a density, *ρ*_0_, of 7.5 g/cm^3^ was selected. The wave velocity, *c*_0_, was 3.57 × 10^5^ cm/s. The front and rear cover plates were made of ASTM 1045 steel and had diameters of 60 mm, densities, *ρ*_1_, of 7.85 g/cm^3^, and wave velocities, *c*_1_ and *c*_2_, of 3.57 × 10^5^ cm/s. The electrode sheet was made of the ASTM C28000 copper alloy and had an outer circle diameter of 60 mm, a density of 8.92 g/cm^3^, and a wave velocity, *c*_3_, of 4.70 × 10^5^ cm/s. Considerations of the asymmetric structure revealed that the rear cover plate, the four piezoelectric ceramic plates, and the electrode plates formed a 1/4-wavelength segment, while the front cover plate also formed a 1/4-wavelength segment. The lengths, *l*_1_ and *l*_2_, of the front and rear cover plates, respectively, can be expressed by Equations (1)–(8), as follows:

(1) tanθ1=Z0Z1T−T+tanθ022T+m3cotθ3,

(2) $$Z_{i}=S_{i}\rho_{i}c_{i}\;\;\;\;\;(i=1,2,3),$$

(3) $$\theta_{i}=\omega l_{i}/c_{i}\;\;\;(i=1,2,3),$$

(4) $$m_{3}=Z_{3}/Z_{0},$$

(5) ω=2πf,

(6) $$T=cot\theta_{0}-\frac{{\left(K_{33}\right)}^{2}}{\theta_{0}},$$

(7) $$l_{1}=\frac{c_{1}\theta_{1}}{\omega },$$

(8) $$l_{2}=\frac{c_{2}}{4f}.$$

In the above equations, *K*_33_ = 0.64, *Z_i_* represents the impedance, *θ_i_* is the phase angle, and ω  denotes the angular velocity.

The rear cover length was calculated to be 29.5 mm and the front cover length was determined to be 65 mm. The amplifying bar was a half-wavelength stepped amplifying bar with a circular cross-section. Its material was the same as that of the front and rear cover plates, the diameter of its large cylinder, *D*_4_, was 60 mm, and its length, *l*_4_, was 65 mm. The small cylinder had a diameter, *D*_5_, of 30 mm and a length, *l*_5_, of 65 mm.

**Figure 2:**
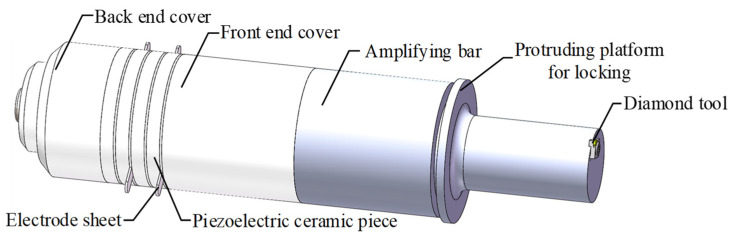
Assembly of the transducer, amplifying bar, and tool.

Based on a specific design size, the NX12.0 software was used to produce drawings of the transducer, the amplifying bar, the electrode sheet, and the tool. It was also used to complete the assembly, as shown in Figure 2. To prevent errors caused by importing other finite element analysis (FEA) software, the NX12.0 pre- and post-processing modules were used to perform a modal analysis and obtain the frequency of the longitudinal vibration. After the LUVP device was manufactured and assembled, a displacement–time curve for the tool nose was obtained by a laser vibrometer. The deviation between the theoretical design frequency and the actual operating frequency were then compared.

#### 2.1.2. Modal Analysis

**Figure 3:**
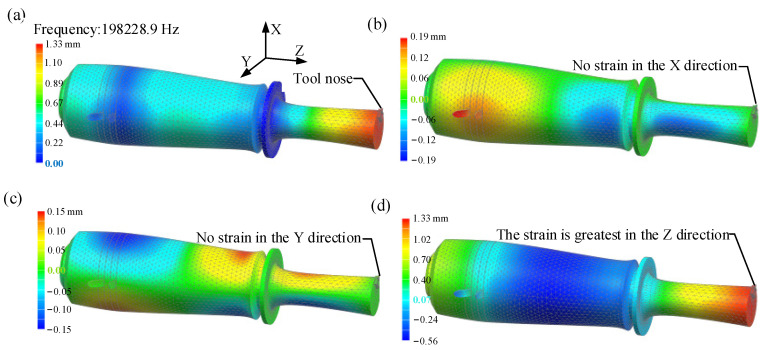
Modal analysis results for the LUVP device: (**a**) three-dimensional displacement cloud image with a resonant frequency of 19.83 kHz, (**b**) cloud image of the displacement in the X-direction, (**c**) cloud image of the displacement in the Y-direction, and (**d**) cloud image of the displacement in the Z-direction.

The modal analysis results for the LUVP device had 10 orders; of these, the seventh resonant frequency, *f_r_*, was 19.83 kHz, which was 3.4% away from the design value. At this resonant frequency, the device vibrated longitudinally, as shown in Figure 3a. Notably, the maximum displacements of the tool nose in the X- and Y-directions were both zero, as shown in Figure 3b and Figure 3c, respectively. In other words, the maximum tool displacement was in the Z-direction, as shown in Figure 3d. The amplitude obtained in the modal analysis was not the actual working amplitude and was only used to analyze the changing trends of the displacement cloud images. The exact working amplitude had to be measured with a laser vibrometer.

#### 2.1.3. Longitudinal Ultrasonic Vibration Characteristics Test

**Figure 4:**
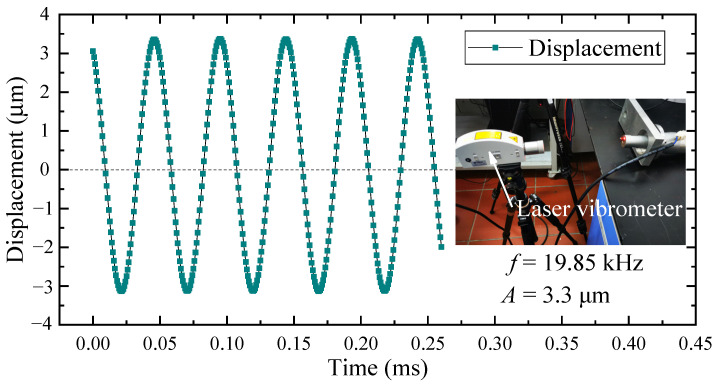
A single-point laser vibrometer was used to obtain a displacement–time curve for the tool nose. The image on the right shows the setup for the tool head vibration characteristics measurements.

The longitudinal vibration characteristics of the tool nose were measured with a laser vibrometer (HSV-E-100-01MC) (Polytec, Karlsruhe, Germany). The measurement results, which are presented in Figure 4, show that the sinusoidal waveform was stable, the amplitude, *A*, was approximately 3.3 μm, and the operating frequency, *f*, was 19.85 kHz. Additionally, there was no noticeable waveform when detecting the transverse vibration characteristics of the tool nose. The deviation between the operating frequency and the modal analysis frequency was 0.1%; therefore, the requirements of the LUVP experiment were met.

### 2.2. Experimental Platform and Materials

**Figure 5:**
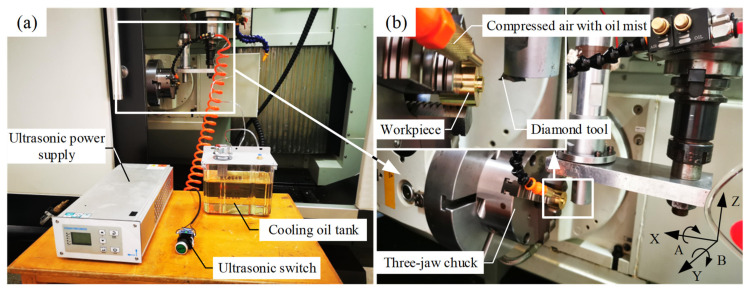
Experimental platform to compare the precision planing and LUVP techniques for processing MPAs: (**a**) the experimental platform, which was built using a five-axis machine tool, and (**b**) the cutting area, which was cooled and lubricated by compressed air that contained an oil mist.

After the LUVP device was assembled, the experimental platform that would be used to compare the planing techniques was built, as shown in Figure 5. The LUVP device was installed on the spindle of the five-axis machine tool (model WN-5V25) (Weino, Putian City, China), which was equipped with an orientation function that enabled the spindle to adopt any turning angle. If the ultrasonic power starting switch was not turned on, precision planing was performed. When the ultrasonic power starting switch was engaged, however, then the LUVP was performed. This starting switch is depicted in Figure 5a. The experimental platform used compressed air that contained an oil mist for cooling and lubrication; this prevented the MPA from becoming contaminated by impurities present in a circulating coolant. The cooling and lubrication regions were both in the cutting area, as shown in Figure 5b.

**Figure 6:**
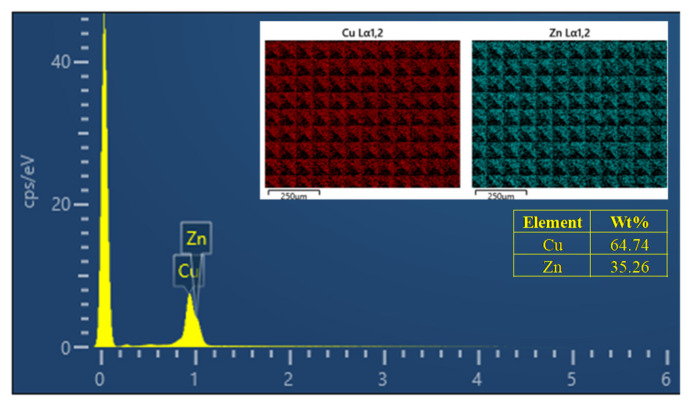
EDS analysis results in local regions of the workpiece. The distributions of copper and zinc elements in the sampling area are shown in the upper-right corner.

Because MTP copper is brass and the C26800 has excellent cutting performance for brass, the C26800 was selected as the workpiece material. Energy-dispersive spectroscopy (EDS), which was performed with a Crossbeam 550 Oxford Xplore30 machine (Zeiss, Oberkochen, Germany), showed that the workpiece had a Cu content of 64.74% and a Zn content of 35.26%. The chemical composition of the workpiece thus met the requirements of the ASTMB36/B36M standard [27], as shown in Figure 6. The workpiece was a stepped shaft; its large cylinder had a diameter of 30 mm and a length of 25 mm, while its small cylinder had a diameter of 10 mm and a length of 5 mm, as shown in the upper-left corner of Figure 5b. Before the experiment, the large cylinder of the workpiece was clamped by a three-jaw chuck and the end face of the small cylinder was milled with a milling cutter. The workpiece was not sanded with sandpaper before the planing comparison test because some of the sand could have fallen off the sandpaper and become embedded in the workpiece surface. The diamond tool could then hit the sand during the planing experiment, which could cause the tool to break, and thereby interfere with the experimental results.

### 2.3. Experimental Scheme for a Single-Factor Comparison between the Precision Planing and LUVP Techniques

**Figure 7:**
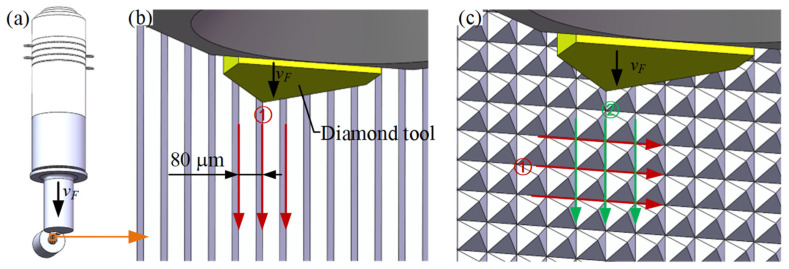
MPA planing path: (**a**) feed speed, *v_F_*, of the tool, which was in the downward direction, (**b**) cutting of the V-shaped grooves in direction ①, and (**c**) cutting of the V-shaped grooves in direction ② after 90° rotation of the workpiece.

When processing an MPA on the end face of the small cylinder of the workpiece, the cutting path used in the precision planing method was the same as that used in the LUVP method; this cutting path is illustrated in Figure 7a. The cutting of the MPA was completed in three steps. In the first step, V-shaped grooves were cut in direction ①; the interval between these grooves was 80 μm, as shown in Figure 7b. In the second step, the workpiece was rotated counterclockwise by 90°. In the third step, V-shaped grooves were cut in direction ②, as shown in Figure 7c. The bottom sides of the micro-pyramids had lengths of 80 μm, and the total heights of the V-shaped grooves were 56.60 μm when the angle was 70.5°. Based on considerations of the milling errors on the small cylindrical face, the total cutting depth for the V-shaped grooves was set to 60 μm. The V-shaped grooves were produced by five separate cuts with sequential cutting depths of 20 μm, 20 μm, 10 μm, 8 μm, and 2 μm, as shown in Table 1.

The LUVP tool vibrated reciprocally, so it cut intermittently. The linear velocity, *v_f_*, of the tool nose vibration was obtained from Equations (9) and (10), and it had values in the −24,620–24,620 mm/min range, as follows:

(9) ω=2πf,

(10) $$v_{f}=2\pi Afsin\left(\omega t\right).$$

In Equation (10), *A* represents the vibration amplitude, which was 3.3 μm, and *t* is the time. To employ the vibration characteristics of the LUVP method, it was necessary to ensure that the minimum cutting speed of the tool nose relative to the workpiece, *v_s_*, was less than 0. According to Equation (11), the feed speed of the machine tool, *v_F_*, must be less than 24,620 mm/min, as follows:

(11) $$v_{s}=v_{F}+v_{f}.$$

The maximum *Z*-axis speed of the selected five-axis machine tool was 2500 mm/min. The feed speed was eventually chosen to be 2000 mm/min to improve the cutting efficiency, as shown in Table 1. The cutting parameters were the same for both the precision planing and LUVP methods; the only difference between the methods was whether the ultrasonic power supply was employed.

### 2.4. Detection Method

**Figure 8:**
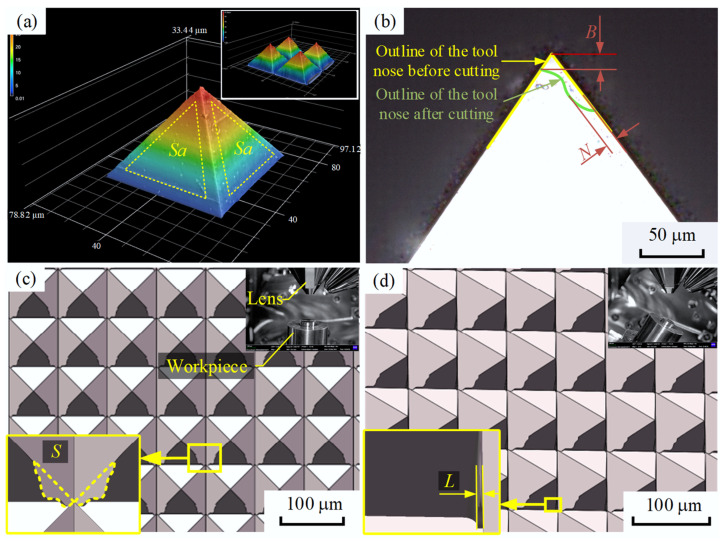
Single-factor test to compare the precision planing and LUVP methods. (**a**) CLSM was used to measure the surface roughness on the MPA sides, *Sa*. (**b**) CLSM was used to measure the nose wear of the diamond tool, *B*, and cutting-edge wear, *N*. (**c**) The MPA morphology was obtained by SEM when the workpiece was in an upright orientation. The upper-right corner depicts the relative positions of the workpiece and the lens. The Image J software was used to analyze the projected area of the edge burrs, *S*, as shown in the lower-left corner. (**d**) The MPA morphology was also obtained by SEM when the workpiece was tilted. The upper-right corner depicts the relative positions of the workpiece and the lens. The measured edge burr thickness, *L*, is shown in the lower-left corner.

Because a diamond molding tool was used, the profile of the tool was directly mapped onto the surface of the workpiece, thereby affecting its surface quality. The surface roughness, tool nose wear, edge burr projection area, and MPA thickness were used as experimental indexes. Confocal laser scanning microscopy (CLSM), which was performed with a VK-X3000 machine (Keyence, Osaka, Japan), was used to measure the surface roughness on the MPA sides, *Sa*, as well as the diamond tool nose wear, *B*, and cutting-edge wear, *N*, as shown in Figure 8a and Figure 8b, respectively. The profiles of the MPAs were measured using scanning electron microscopy (SEM), which was performed by a Crossbeam 550 machine. The overall topography of the MPAs was obtained when the workpiece was placed in an upright orientation. The projected area of the edge burrs, *S*, was obtained using the Image J 1.8.0 software, as shown in Figure 8c. The edge burr thickness, *L*, was obtained when the workpiece was placed at a tilt, as shown in Figure 8d.

## 3. Results

### 3.1. Surface Roughness and Diamond Tool Nose Wear

**Figure 9:**
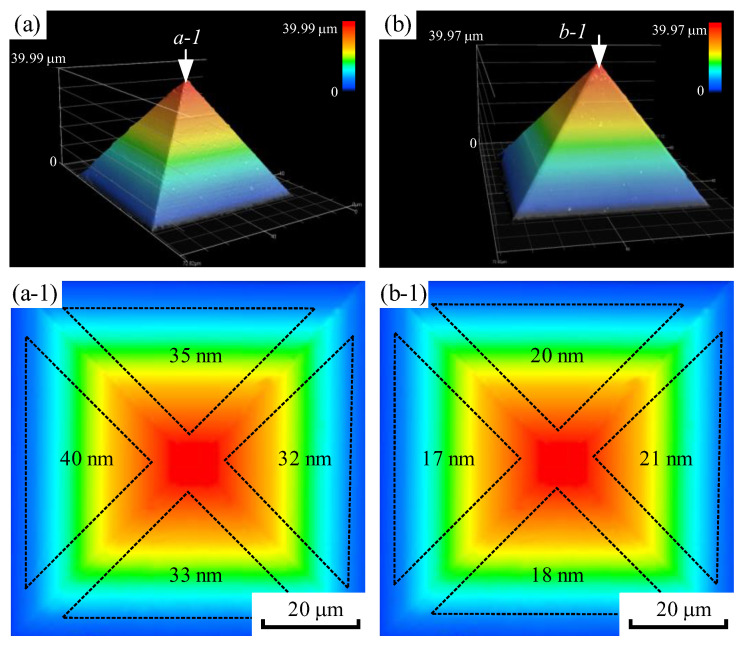
Comparison of the side surface roughness values of micro-pyramids produced by the precision planing and LUVP methods: (**a**) CLSM image of a precision-planed micro-pyramid, (**b**) CLSM image of a micro-pyramid produced by LUVP, (**a-1**) observation of the micro-pyramid in (**a**) along the (**a-1**) direction, and (**b-1**) observation of the micro-pyramid in (**b**) along the (**b-1**) direction. Each value in (**a-1**,**b-1**) is the *Sa* value for the corresponding area within the black dashed lines.

**Figure 10:**
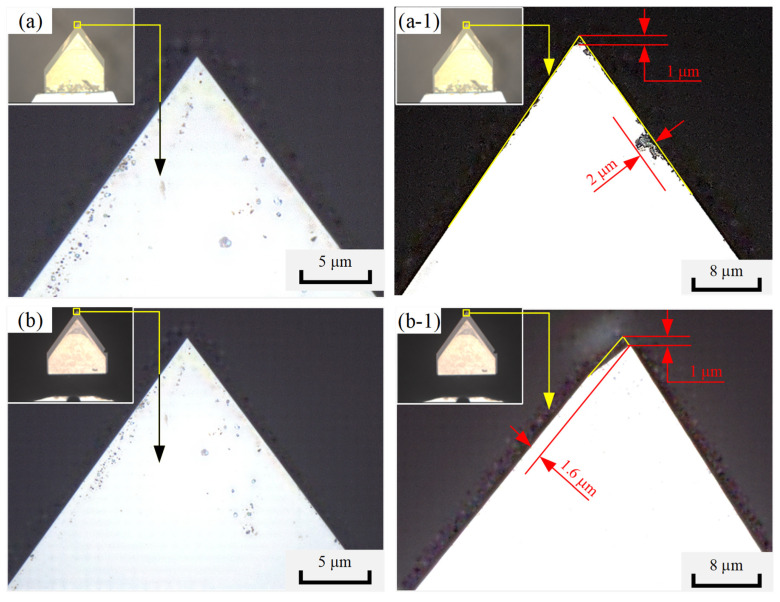
CLSM images of the diamond tools: (**a**) diamond tool before precision planing, (**a-1**) diamond tool after precision planing, (**b**) diamond tool before LUVP, and (**b-1**) diamond tool after LUVP.

The results of the single-factor experiment show that the average surface roughness of the four sides of a micro-pyramid processed by precision planing was approximately 35 nm. In contrast, the average surface roughness of a micro-pyramid produced by LUVP was approximately 19 nm. These results demonstrate that the surface roughness of the LUVP micro-pyramids was smaller than that of the micro-pyramids produced by precision planing, as shown in Figure 9. The tool nose wear that occurred during the precision planing process was approximately 1.0 μm, and the cutting-edge wear was approximately 2.0 μm. The tool nose wear that occurred during the LUVP process was approximately 1.0 μm, and the cutting-edge wear was approximately 1.6 μm. Thus, the LUVP process generated slightly less tool wear compared to the precision planing process, as shown in Figure 10.

### 3.2. Edge Burr Projection Area and Thickness

**Figure 11:**
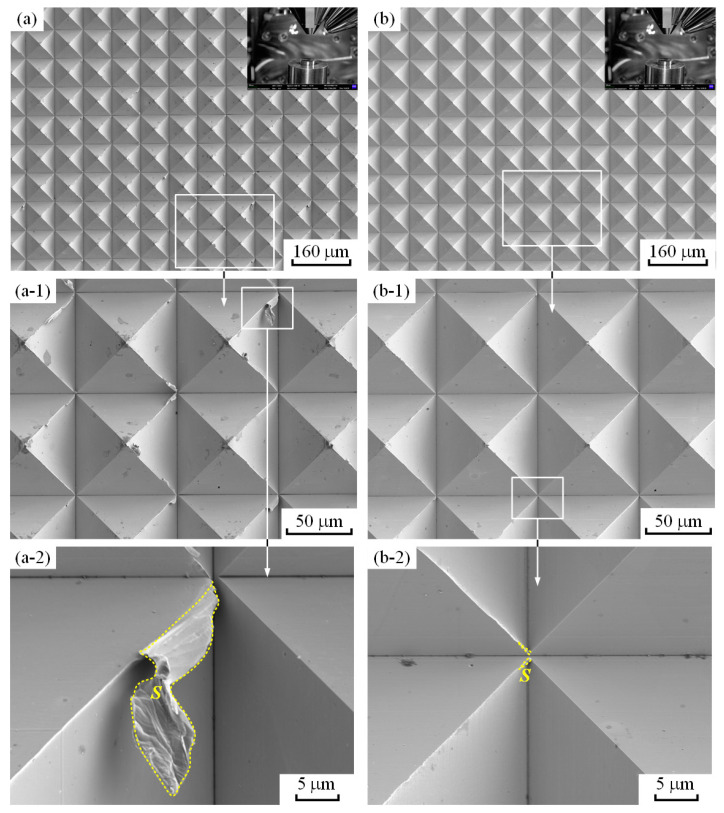
SEM images of MPAs generated by precision planing and LUVP: (**a**) precision-planed MPA (the top-right corner depicts the relative positions of the lens and the workpiece), (**b**) LUVP MPA (the top-right corner depicts the relative positions of the lens and the workpiece), (**a-1**,**b-1**) partial enlargements of (**a**) and (**b**), respectively, and (**a-2**,**b-2**) partial enlargements of (**a-1**,**b-1**), respectively. The areas enclosed by dotted yellow lines are projected edge burr areas. The projected areas of the edge burrs in (**a-2**,**b-2**) were calculated using the Image J software.

The SEM measurement results demonstrate that the MPA morphologies produced by the precision planing and LUVP methods were visible and that no corrugated surface was found, as shown in Figure 11a,b. Significantly fewer and smaller edge burrs were present on the MPA processed by LUVP than on that processed by precision planing, as shown in Figure 11(a-1,b-1). The average projected area of the edge burrs, *S*, on the precision-planed MPA was 53 μm^2^, as shown in Figure 11(a-2), while it was 0.23 μm^2^ on the MPA formed by LUVP, as shown in Figure 11(b-2). In general, the projected areas of the edge burrs on the LUVP MPA were significantly smaller than those on the precision-planed MPA.

### 3.3. Edge Burr Thickness

**Figure 12:**
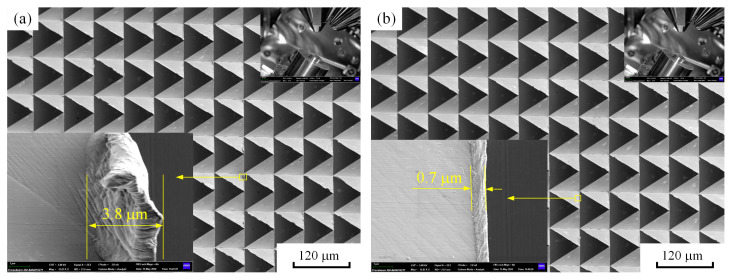
SEM edge burr thickness measurements for MPAs processed by the precision planing and LUVP methods: (**a**) edge burr thicknesses for the precision-planed MPA and (**b**) edge burr thicknesses for the LUVP MPA.

After the workpiece in the SEM sample chamber was tilted by 35.25°, the edge burr thickness was analyzed. The maximum edge burr thickness, *L*, of the precision-planed MPA was approximately 3.8 μm, as shown in Figure 12a, while it was approximately 0.7 μm for the LUVP MPA, as shown in Figure 12b. In general, the thicknesses of the edge burrs on the MPA processed by LUVP were significantly smaller than on that processed by precision planing. 

## 4. Discussion

### 4.1. Vibration Analysis

**Figure 13:**
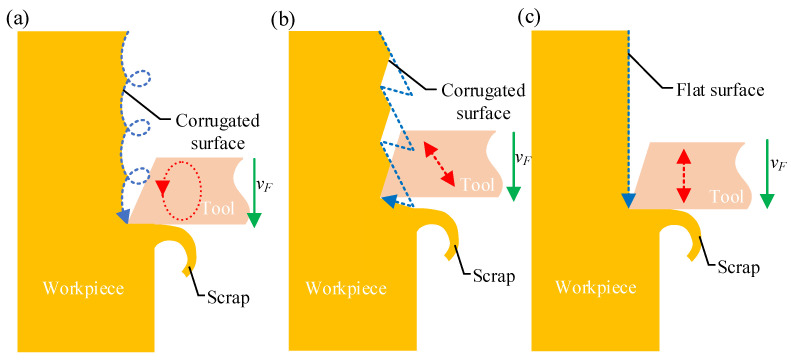
Schematic diagram of ultrasonic planing with different vibration forms: (**a**) elliptical vibration, (**b**) longitudinal vibration that is not parallel to the feed direction, and (**c**) longitudinal vibration in a direction parallel to the feed direction. The red line represents the vibration direction of the tool nose, the green line illustrates the feed direction of the tool nose, and the blue line represents the trajectory of the tool nose.

The tool nose exhibits two kinds of motion, vibration and feed, during ultrasonic vibration-assisted planing. When the vibration direction is not aligned with the feed direction, the planing of the workpiece produces a corrugated surface, as shown in Figure 13a,b. When the vibration direction is parallel to the feed direction, the planing of the workpiece produces a flat surface, as shown in Figure 13c. To prevent the formation of corrugation on the workpiece surface, the vibration direction of the LUVP device must be parallel to the direction of the tool feed. The vibration characteristics of the LUVP device indicate that its operating vibration frequency, *f*, is very similar to the resonant frequency, *f_r_*, obtained during the modal analysis, as shown in Figure 3 and Figure 4. According to the modal analysis results, the tool nose vibration is a longitudinal reciprocating vibration and vibration occurs in no other direction, as shown in Figure 7. When the LUVP device was operating, the tool nose vibration displacement–time curve was sinusoidal, which indicates that the vibration effect was good, as shown in Figure 4. Therefore, the LUVP device met the experimental requirements.

### 4.2. Surface Roughness and Tool Nose Wear

**Figure 14:**
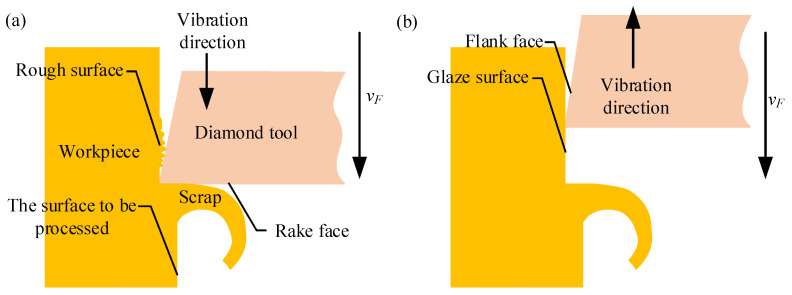
Schematic diagram of the LUVP process: (**a**) tool vibration that occurs in the same direction as the tool feed and (**b**) tool vibration that occurs in the direction opposite to that of the tool feed.

During LUVP, when the tool vibration occurs in the tool feed direction, the actual cutting speed increases, while the cutting force and the material deformation both decrease [28,29]; these changes are conducive to the removal of edge burrs, as shown in Figure 14a. When the tool vibration occurs in the direction opposite to that of the tool feed, the actual cutting direction changes. Thus, the tool moves backward relative to the workpiece. During this process, the flank face of the tool produces an ironing effect on the machined surface of the workpiece [30,31] that improves the surface quality, as shown in Figure 14b.

During the LUVP conducted during this study, periodic cutting with an amplitude of 3.3 μm was performed. According to Equation (10), the linear velocity of the tool nose vibration, *v_f_*, was in the −24,620–24,620 mm/min range. The tool feed speed, *v_F_*, was 2000 mm/min. According to Equation (11), the cutting speed, *v_s_*, of the tool nose relative to the workpiece was in the −22,620–24,620 mm/min range, and the maximum cutting speed was much greater than the precision planing cutting speed of 2000 mm/min. Because the LUVP cutting speed was large, the cutting force was small; thus, the workpiece deformation was small. Additionally, the ironing effect produced by the tool flank face on the machined surface during the tool vibration process caused the surface roughness of the MPA machined by LUVP to be smaller than that of the MPA processed by precision planing.

During the LUVP process, when *v*_s_. was −22,620–0 mm/min, the rake face of the diamond tool did not participate in cutting; rather, the flank face ironed the machined surface. When *v*_s_. was 0–24,620 mm/min, the rake face of the diamond tool cut the workpiece. Due to the high working frequency of the LUVP method, which was 19.85 kHz, the workpiece was ironed by the flank face of the diamond tool up to 19,850 times each second; this ironing readily generated micro-wear on the diamond tool nose. Therefore, compared with precision planing, although LUVP exerts smaller cutting forces, it still causes tool wear.

### 4.3. Edge Burrs

**Figure 15:**
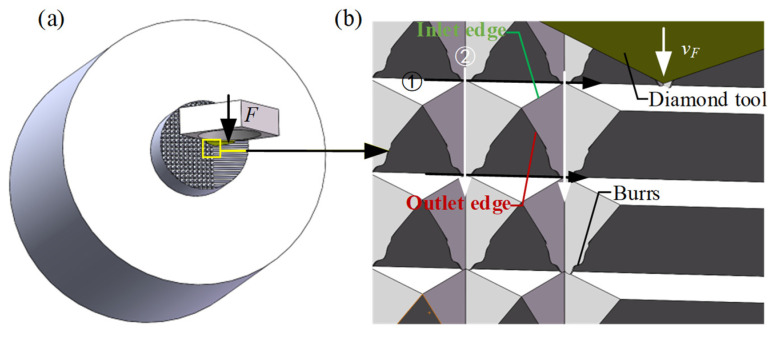
The forming of edge burrs during planing of an MPA: (**a**) diamond tool planing of an MPA and (**b**) partial enlargement of (**a**). ① and ② represent the tool paths used during the MPA cutting.

The burrs on an MPA were concentrated on the edges of the cutting outlets. When the V-shaped grooves were cut in direction ①, continuous cutting occurred with no burr generation. After the workpiece was rotated 90°, when the V-shaped grooves were cut in direction ②, the cutting was intermittent, as shown in Figure 15. Specifically, during the formation of each micro-pyramid, periodic cutting into and out of the material occurred. The entrance edges formed by cutting into the material had no burrs; however, when the tool cut out of the material, burrs were generated at the outlet edges due to plastic deformation of the material, as shown in Figure 15b.

**Figure 16:**
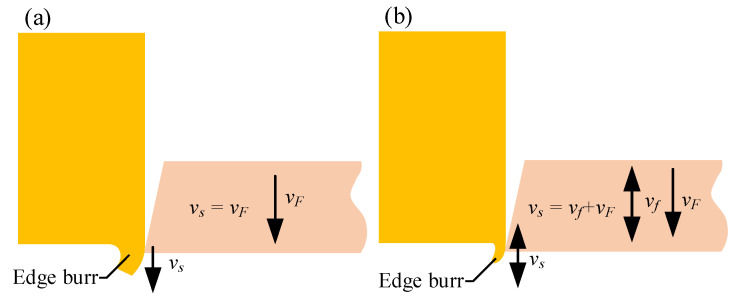
Edge burr formation process at the cutting outlet: (**a**) precision planing (*v_s_* = *v_F_*) produced large cutting-outlet edge burrs and (**b**) LUVP (*v_s_* = *v_f_* + *v_F_*) produced small cutting-outlet edge burrs.

The edge burr projected area and thickness analyses revealed that the sizes and numbers of the edge burrs on the micro-pyramids processed by LUVP were significantly smaller than those on the micro-pyramids processed by precision planing. This result occurred because the LUVP tool cut 19,850 times per second, and the maximum actual value cutting speed during LUVP was much greater than that during precision planing. Therefore, despite the small cutting force [32], there was minimal material plastic deformation and fewer edge burrs, as shown in Figure 16.

## 5. Conclusions

To improve the surface quality of MPAs, reduce the sizes and numbers of edge burrs, and prevent the generation of corrugated surfaces, the LUVP method for processing MPAs is proposed in this paper. The results of a single-factor experiment that was conducted to compare the precision planing and LUVP methods for processing MPAs are also presented. The study produced the following three primary conclusions:

- The results of a modal analysis and a vibration characteristics test indicated that the designed LUVP device had an operating frequency of 19.85 kHz, an amplitude of 3.3 μm, and longitudinal vibration that was straight and parallel to the feed direction. Thus, it met the experimental requirements for the LUVP processing of MPAs.
- No corrugation was produced on the workpiece surfaces in either the LUVP or precision planing processes. The lateral surface roughness of the MPA processed by LUVP was smaller than that of the MPA produced by precision planing, and the LUVP process generated slightly less tool wear than the precision planing process.
- Analyses of the edge burr projected area and thickness revealed that the sizes and number of edge burrs on the LUVP MPA were significantly smaller than those on the precision-planed MPA. This research lays a foundation for developing micro-pyramid plastic films with greater precision.

## Figures and Tables

**Table 1:** Cutting parameters.

Feed Speed (mm/min)	Cutting Depth of Five Cuts (μm)				
	First Time	Second Time	Third Time	Fourth Time	Fifth Time
2000	20	20	10	8	2

## Data Availability

The original contributions presented in the study are included in the article. Further inquiries can be directed to the corresponding authors.

## References

[1] Kim J., Kim B., Kang R., Lee M., Lee B., Kim S. (2024). High-efficiency upright solar panels with antireflective microprism-imprinted sheets. Cell Rep. Phys. Sci..

[2] Ge S., Liu W., Zhou S., Li S., Sun X., Huang Y., Yang P., Zhang J., Lin D. (2018). Design and preparation of a micro-pyramid structured thin film for broadband infrared antireflection. Coatings.

[3] Xu P., Luo T., Zhang X., Su Z., Huang Y., Li X., Zou Y. (2018). Design and optimization of a partial integrated backlight module. Opt. Commun..

[4] Qu C., Xu Y., Xiao Y., Zhang S., Liu H., Song G. (2021). Multifunctional displays and sensing platforms for the future: A review on flexible alternating current electroluminescence devices. ACS Appl. Electron. Mater..

[5] Lee H., Yi A., Choi J., Ko D., Jung Kim H. (2022). Texturing of polydimethylsiloxane surface for anti-reflective films with super-hydrophobicity in solar cell application. Appl. Surf. Sci..

[6] Saxe S.G. (1989). Prismatic film light guides: Performance and recent developments. Sol. Energy Mater..

[7] Mou X., Lu Y., Mo R., Sun J. (2024). Precision grinding of ceramics and ceramic-matrix composites surfaces with controllable microarray structures. Mater. Today Commun..

[8] Gao J., Xu Z., Lei Y., Huang S. (2024). Fly-cutting processing of micro-triangular pyramid arrays and synchronous micro-scrap removal. Micromachines.

[9] Wu B., Zong W. (2022). A modified diamond micro chiseling method for machining large scale retroreflective microstructure on nickel phosphorus alloy. J. Mater. Process. Technol..

[10] Zhang G., Ma Y., Luo T., Cao S., Huang Z. (2023). Fabrication of hierarchical micro-groove structures by vibration assisted end fly cutting. J. Mater. Process. Technol..

[11] Zhang P., Jing Z., Goel S., Hou X., Wang C., Cheung C.F., Tian Y., Guo J. (2024). Theoretical and experimental investigations on conformal polishing of microstructured surfaces. Int. J. Mech. Sci..

[12] Joao D., Rangel O., Milliken N., Tutunea-Fatan O.R., Bordatchev E. (2022). Single-flank machining strategy for ultraprecise single-point cutting of v-grooves. Int. J. Adv. Manuf. Technol..

[13] Li H., Xu Z., Pi J., Zhou F. (2020). Precision cutting of the molds of an optical functional texture film with a triangular pyramid texture. Micromachines.

[14] Zhang S., Zhou Y., Zhang H., Xiong Z., To S. (2019). Advances in ultra-precision machining of micro-structured functional surfaces and their typical applications. Int. J. Mach. Tools Manuf..

[15] Li H., Xu Z. (2021). Micro-burrs in machining original molds of an optical functional film with a triangular pyramidal texture. J. Micromech. Microeng..

[16] Jiang T., Yang J., Pi J., Luo W., Zhang J. (2022). Experimental and analytical study of ultrasonic elliptical vibration cutting of micro-pyramid reflective mold based on guided wave transmission. Int. J. Adv. Manuf. Technol..

[17] Han X., Zhang D. (2020). Effects of separating characteristics in ultrasonic elliptical vibration-assisted milling on cutting force, chip, and surface morphologies. Int. J. Adv. Manuf. Technol..

[18] Wei S., Zou P., Zhang J., Duan J., Fang R. (2022). Theoretical and experimental research on 3d ultrasonic vibration–assisted turning driven by a single actuator. Int. J. Adv. Manuf. Technol..

[19] Gao G., Xia Z., Su T., Xiang D., Zhao B. (2021). Cutting force model of longitudinal-torsional ultrasonic-assisted milling ti-6al-4v based on tool flank wear. J. Mater. Process. Technol..

[20] Xie Z., Liu Z., Han L., Wang B., Xin M., Cai Y., Song Q. (2022). Optimizing amplitude to improve machined surface quality in longitudinal ultrasonic vibration-assisted side milling 2.5d c/sic composites. Compos. Struct..

[21] Gao H., Ma B., Zhu Y., Yang H. (2022). Enhancement of machinability and surface quality of ti-6al-4v by longitudinal ultrasonic vibration-assisted milling under dry conditions. Measurement.

[22] Liu Y., Ma L., Liu F., Fu B., Yao J. (2023). A novel model of vibration plowing effect for longitudinal ultrasonic vibration-assisted drilling. J. Manuf. Process..

[23] Li Y., Jiao F., Zhang Z., Wang X., Niu Y. (2023). Mechanical drilling force model for longitudinal ultrasonic vibration-assisted drilling of unidirectional cfrp. J. Mater. Process. Technol..

[24] Zhao M., Xue B., Li B., Zhu J., Wenbinsong, Nie L. (2024). Modeling of grinding force in longitudinal ultrasonic vibration–assisted grinding alumina ceramics and experimental evaluation. Int. J. Adv. Manuf. Technol..

[25] Duan Z., Chen T., Li H., Zhang C., Liu F. (2023). Longitudinal ultrasonic vibration effects on grinding mechanism in side and end grinding of 2.5d cf/sic composites. Int. J. Adv. Manuf. Technol..

[26] Qian C., Tian Y., Ahmad S., Ma Z., Li L., Fan Z. (2024). Theoretical and experimental investigation on magnetorheological shear thickening polishing force using multi-pole coupling magnetic field. J. Mater. Process. Technol..

[27] (2023). Standard Specification for Brass Plate, Sheet, Strip, and Rolled Bar.

[28] Cao Y., Ding W., Zhao B., Wen X., Li S., Wang J. (2022). Effect of intermittent cutting behavior on the ultrasonic vibration-assisted grinding performance of inconel718 nickel-based superalloy. Precis. Eng..

[29] Zhao G., Xin L., Li L., Zhang Y., He N., Hansen H.N. (2023). Cutting force model and damage formation mechanism in milling of 70wt% si/al composite. Chin. J. Aeronaut..

[30] Zhou J., Lu M., Lin J., Wei W. (2023). Influence of tool vibration and cutting speeds on removal mechanism of sicp/al composites during ultrasonic elliptical vibration-assisted turning. J. Manuf. Process..

[31] Peng Z., Zhang X., Zhang D. (2021). Integration of finishing and surface treatment of inconel 718 alloy using high-speed ultrasonic vibration cutting. Surf. Coat. Technol..

[32] Peng Z., Zhang X., Zhang D. (2021). Effect of radial high-speed ultrasonic vibration cutting on machining performance during finish turning of hardened steel. Ultrasonics.

